# Optimal hydration volume among high-risk patients with advanced congestive heart failure undergoing coronary angiography

**DOI:** 10.18632/oncotarget.25315

**Published:** 2018-05-04

**Authors:** Shi-Qun Chen, Yong Liu, Wei Jie Bei, Ying Wang, Chong-Yang Duan, Deng-Xuan Wu, Kun Wang, Ping Yan Chen, Ji-Yan Chen, Ning Tan, Li-Wen Li

**Affiliations:** ^1^ Department of Cardiology, Guangdong Provincial Key Laboratory of Coronary Heart Disease Prevention, Guangdong Cardiovascular Institute, Guangdong General Hospital, Guangdong Academy of Medical Sciences, Guangzhou, Guangdong, China; ^2^ Department of Cardiology, Guangdong General Hospital Zhuhai Hospital (Zhuhai Golden Bay Center Hospital), Zhuhai, Guangdong, China; ^3^ The George Institute for Global Health, Sydney, Australia; ^4^ National Clinical Research Center for Kidney Disease, State Key Laboratory of Organ Failure Research, Department of Biostatistics, School of Public Health and Tropical Medicine Southern Medical University, Guangzhou, Guangdong, China

**Keywords:** heart failure, acute kidney injury, hydration, contrast, coronary angiography

## Abstract

We investigated the relationship between weight-adjusted hydration volumes and the risk of developing contrast-induced acute kidney injury (CI-AKI) and worsening heart failure (WHF) and explored the relative safety of optimal hydration volumes in patients with advanced congestive heart failure (CHF) undergoing coronary angiography (CAG) or percutaneous coronary intervention. We included 551 patients with advanced CHF (New York Heart Association class > 2 or history of pulmonary edema) undergoing CAG (follow-up period 2.62 ± 0.9 years). There was a significant association between hydration volume-to-weight ratio (HV/W) (quintile Q1, Q2, Q3, Q4, and Q5) and the incidence of CI-AKI (3.7%, 14.6%, 14.3%, 21.1%, and 31.5%, respectively) and WHF (3.6%, 5.4%, 8.3%, 13.6%, and 19.1%, respectively) (all *P-trend* < 0.001). Receiver operating curve analysis indicated that HV/W = 15 mL/kg and the mean HV/W (60.87% sensitivity and 64.96% specificity) were fair discriminators for CI-AKI (C-statistic 0.696). HV/W >15 mL/kg independently predicted CI-AKI (adjusted odds ratio [OR] 2.33; *P* = 0.016) and WHF (adjusted OR 2.13; *P* = 0.018). Moreover, both CI-AKI and WHF were independently associated with increased long-term mortality. Thus, for high-risk patients with advanced CHF undergoing CAG, HV/W > 15 mL/kg might be associated with an increased risk of developing CI-AKI and WHF. The potential benefits of a personalized limitation of hydration volume need further evaluation.

## INTRODUCTION

Contrast-induced acute kidney injury (CI-AKI), a common complication following coronary angiography (CAG) or percutaneous coronary intervention (PCI), is associated with in-hospital and long-term mortality, especially among high-risk patients such as those with congestive heart failure (CHF) [1, 2]. Adequate hydration is used as a basic and effective strategy for the prevention of CI-AKI among patients undergoing CAG or PCI, including patients with advanced CHF (as defined by a New York Heart Association [NYHA] functional classification greater than 2). Furthermore, it has been recommended that the speed of fluid administration be reduced to 0.5 mL/kg/h for patients with heart failure to avoid fluid overload and in-hospital worsening heart failure (WHF), which is associated with poorer short- and long-term outcomes [3]. However, there is no consensus on the optimal hydration volume for the prevention of CI-AKI in patients with advanced CHF [4, 5].

Patients with multiple comorbidities (heart, renal, and liver dysfunction) have a higher risk of fluid overload [6]. Fluid overload is an independent risk factor for acute kidney injury and mortality in critically ill patients [7–9]. Although it has been suggested that hydration rates be adjusted by body weight (mL/kg/h) [10–12], there is a paucity of studies on the optimal hydration volume for avoiding fluid overload in patients with a high risk of WHF and CI-AKI.

In the present study, we investigated the relationship between weight-adjusted hydration volumes and the risk of developing CI-AKI and WHF and explored the relative safety of optimal hydration volumes in patients with advanced CHF undergoing CAG or PCI.

## RESULTS

**Figure 1:**
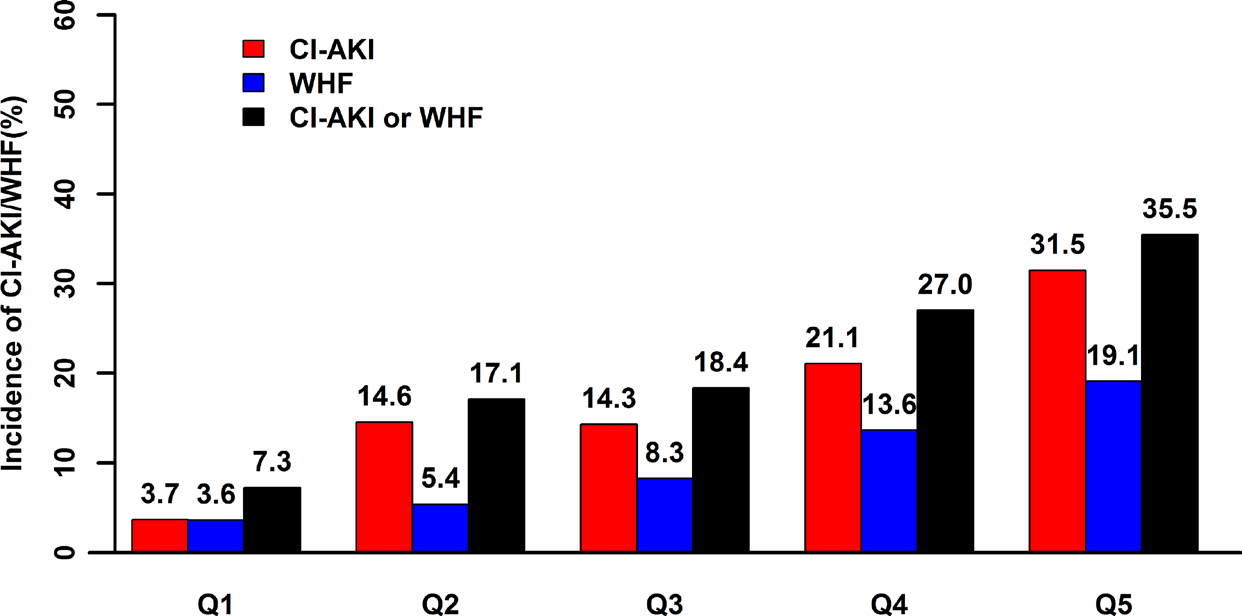
Relationship between HV/W and CI-AKI and WHF The association between HV/W and the percentage of patients with CI-AKI and/or WHF following CAG was significant (*P-trend* < 0.001). HV/W, hydration volume-to-weight (mL/kg); CI-AKI, contrast-induced acute kidney injury; WHF, worsening heart failure.

**Figure 2:**
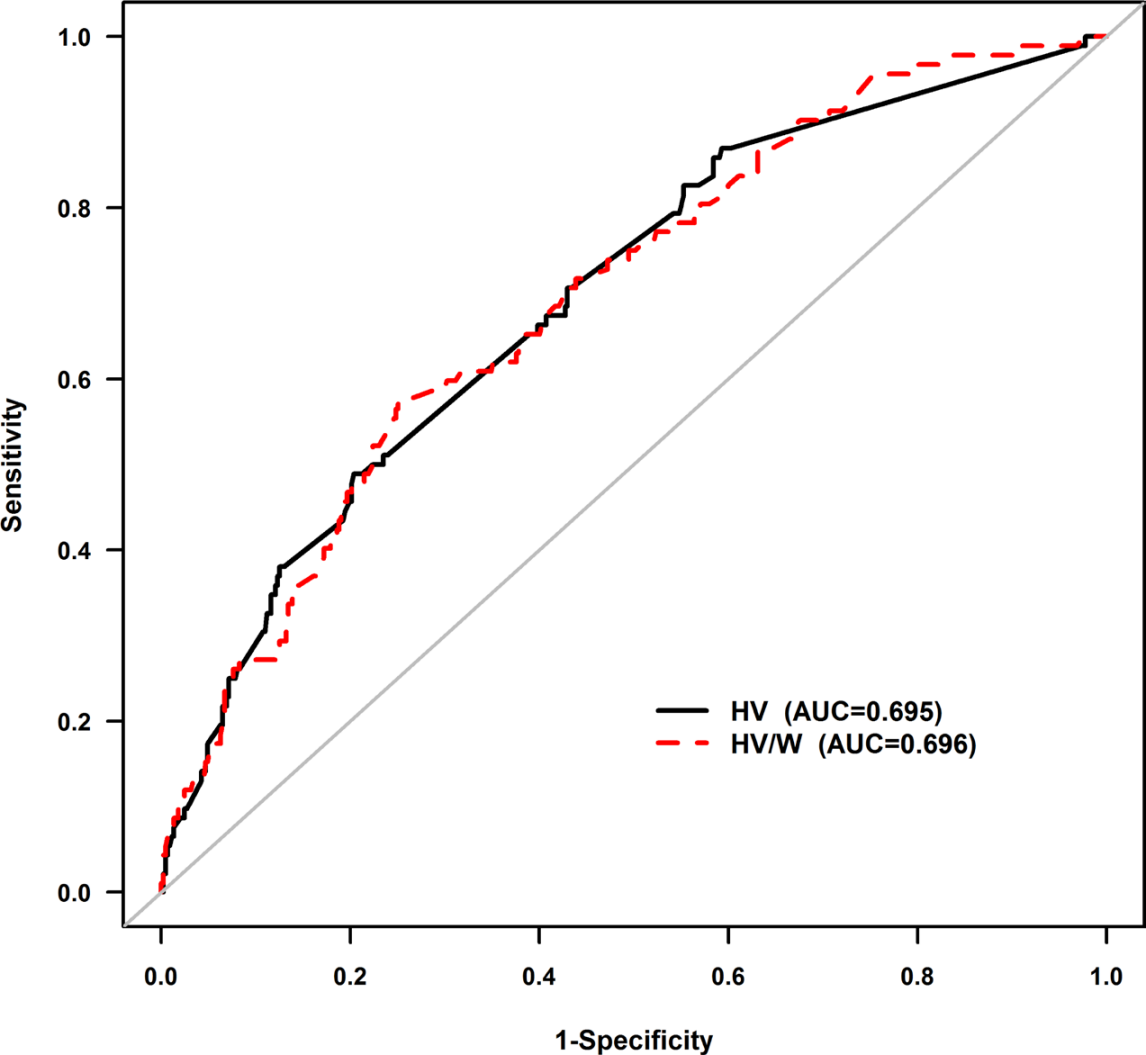
Receiver-operating characteristic curve analysis of the association between HV/W and CI-AKI At a cut-off value of > 15 mL/kg, HV/W exhibited 60.87% sensitivity and 64.96% specificity for detecting CI-AKI. The C-statistic was 0.696. HV/W, hydration volume-to-weight (mL/kg); CI-AKI, contrast-induced acute kidney injury.

A total of 551 patients with advanced CHF (27.4% female; age 66 ± 11 years) were included in the final analysis (Supplementary Figure 1). There was a significant association between hydration volume-to-weight ratio (HV/W) (quintile Q1 [< 8.1 mL/kg, *n* = 110], Q2 [8.1–10.64 mL/kg, *n* = 111], Q3 [10.65–14.75 mL/kg, *n* = 109], Q4 [14.75–20 mL/kg, *n* = 111] and Q5 [> 20 mL/kg, *n* = 110]) and the incidence of CI-AKI (3.7%, 14.6%, 14.3%, 21.1%, and 31.5%, respectively) and WHF (3.6%, 5.4%, 8.3%, 13.6%, and 19.1%, respectively) (all *P-trend* < 0.001) (Figure 1). Receiver operating characteristic (ROC) curve analysis showed a correlation between HV/W ratio and CI-AKI, with a C-statistic of 0.696. In particular, the analysis showed that at a cut-off level of > 15 mL/kg and at the mean value of HV/W, the HV/W ratio exhibited 60.87% sensitivity and 64.96% specificity for predicting CI-AKI (Figure 2).

### Characteristics of patients with HV/W > 15 mL/kg and HV/W ≤ 15 mL/kg

The patients in the HV/W > 15 mL/kg group were significantly older, had higher log B-type natriuretic peptide (BNP) levels and poorer renal function, were more likely to undergo emergency PCI, had more hypotensive episodes and coronary artery lesions, had higher Mehran risk scores [2], and more frequently required diuretics (Table 1). However, there were no significant inter-group differences with respect to diabetes mellitus, anemia, and contrast volume.

**Table 1:** Baseline patient and procedural characteristics according to the hydration volume-to-body weight ratio (HV/W ≤ 15 and HV/W > 15 mL/kg)

Characteristics	Total (*n* = 551)	HV/W		
		HV/W ≤ 15 mL/kg (*n* = 334)	HV/W > 15 mL/kg (*n* = 217)	*P*-value
Age, years	66.42 ± 10.84	64.68 ± 10.89	69.09 ± 10.24	<0.001
Age >75 y, n (%)	126 (22.9)	57 (17.1)	69 (31.8)	<0.001
Female sex, n (%)	151 (27.4)	88 (26.3)	63 (29.0)	0.490
CrCl, mL/min	60.84 ± 27.60	68.71 ± 26.25	48.73 ± 25.19	<0.001
**CrCl ≥60 mL/min, *n* (%)**	249 (45.2)	195 (58.4)	54 (24.9)	<0.001
Serum creatinine, μmol/L	106.21 ± 49.27	93.98 ± 38.08	125.03 ± 57.95	<0.001
Systolic blood pressure	125.93 ± 23.34	126.30 ± 21.41	125.36 ± 26.09	0.658
LVEF, %	51.16 ± 13.60	52.21 ± 14.02	49.54 ± 12.79	0.030
LVEF <40%, n (%)	110 (21.5)	67 (21.5)	43 (21.5)	0.991
Mehran Score	10.79 ± 4.20	9.70 ± 3.53	12.48 ± 4.58	<0.001
Weight, kg	63.43 ± 10.71	65.27 ± 10.53	60.59 ± 10.38	<0.001
**Medical history, *n* (%)**				
Diabetes mellitus	66 (30.1)	93 (27.8)	73 (33.6)	0.147
Smoker	204 (37.0)	122 (36.5)	82 (37.8)	0.765
Hypertension	334 (60.6)	196 (58.7)	138 (63.6)	0.249
Hyperlipidemia	73 (13.2)	49 (14.7)	24 (11.1)	0.222
Prior MI	55 (10.0)	27 (8.1)	28 (12.9)	0.065
History of CABG	6 (1.1)	2 (0.6)	4 (1.8)	0.169
**Laboratory measurements**				
LDL-C, mmol/L	2.82 ± 0.97	2.75 ± 0.96	2.93 ± 0.98	0.118
HDL-C, mmol/L	0.88 ± 0.30	2.93 ± 0.98	0.90 ± 0.34	0.489
Total cholesterol, mmol/L	4.56 ± 1.19	4.52 ± 1.20	4.52 ± 1.20	0.480
logBNP,	7.23 ± 1.65	6.99 ± 1.61	7.66 ± 1.62	<0.001
HbA1c, %	6.76 ± 1.52	6.78 ± 1.58	6.72 ± 1.43	0.699
hs-CRP, mg/L	6.76 ± 1.52	24.72 ± 37.11	28.95 ± 38.98	0.347
Anemia, *n* (%)	224 (41.1)	118 (35.8)	106 (49.3)	0.002
Hematocrit, %	0.38 ± 0.06	0.38 ± 0.05	0.37 ± 0.06	0.024
**Medication, *n* (%)**				
ACEI/ARB	463 (84.0)	289 (86.5)	174 (80.2)	0.047
β-blocker	414 (75.3)	463 (84.0)	154 (71.0)	0.059
CCB	71 (13.0)	35 (10.5)	35 (10.5)	0.035
Diuretics	270 (49.0)	145 (43.4)	125 (57.6)	0.001
**Procedure**				
Emergency PCI, *n* (%)	171 (31.0)	86 (25.7)	85 (39.2)	<0.001
Coronary lesions	2.25 ± 1.07	2.13 ± 1.07	2.45 ± 1.04	0.002
Coronary stents	1.57 ± 1.20	2.45 ± 1.04	2.45 ± 1.04	0.199
Total length of stent (mm)	40.00 ± 32.81	37.44 ± 31.01	44.39 ± 35.36	0.030
Procedure duration (min)	79.37 ± 43.35	76.69 ± 41.46	83.52 ± 45.91	0.073
Contrast volume (mL)	128.40 ± 62.39	127.07 ± 61.56	130.46 ± 63.74	0.533
Contrast volume >200 mL	75 (13.6)	43 (12.9)	32 (14.7)	0.531
HV/W, mL/kg	15.26 ± 9.55	9.61 ± 2.65	23.95 ± 9.80	<0.001
HV, mL	938.75 ± 563.10	619.58 ± 183.42	1430.02 ± 596.27	<0.001

Abbreviations: CrCl, creatinine clearance; LVEF, left ventricular ejection fraction; MI, myocardial infarction; CABG, coronary artery bypass grafting; LDL-C, low-density lipoprotein-cholesterol; HDL-C, high-density lipoprotein-cholesterol; BNP, B-type natriuretic peptide; HbA1c, glycated hemoglobin; hs-CRP, high-sensitivity C-reactive protein; ACEI/ARB, angiotensin-converting enzyme inhibitor/angiotensin receptor blocker; CCB, calcium channel blocker; PCI, percutaneous coronary intervention; HV, hydration volume.

### Outcomes among patients with HV/W > 15 mL/kg and HV/W ≤ 15 mL/kg

CI-AKI incidence was significantly higher in the HV/W > 15 mL/kg than in the HV/W ≤ 15 mL/kg group (26.3% vs 11.0%, *P* < 0.001); the same trend was noted for WHF (16.7% vs 5.7%, *P* < 0.001, Table 2).

**Table 2:** In-hospital and clinical outcomes during follow-up according to the hydration volume-to-body weight ratio (HV/W ≤ 15 and HV/W > 15 mL/kg)

Characteristics	Total (*n* = 551)	HV/W		
		HV/W ≤ 15 mL/kg (*n* = 334)	HV/W > 15 mL/kg (*n* = 217)	*P*-value
**In-hospital outcomes**				
CI-AKI, *n* (%)	92 (17.0)	36 (11.0)	56 (26.3)	<0.001
WHF, *n* (%)	55 (10.0)	19 (5.7)	36 (16.7)	<0.001
**2-year clinical outcomes**				
MACE, *n* (%)	151 (34.2)	71 (26.5)	80 (46.0)	<0.001
Mortality, *n* (%)	66 (15.1)	24 (9.1)	42 (24.6)	<0.001
Dialysis, *n* (%)	17 (4.0)	5 (1.9)	12 (7.5)	0.004
Re-hospitalization, *n* (%)	77 (18.8)	45 (17.3)	32 (21.5)	0.299
TVR, *n* (%)	6 (1.5)	1 (0.4)	5 (3.3)	0.017
Stroke, *n* (%)	7 (1.7)	1 (0.4)	6 (3.9)	0.007
**3-year clinical outcomes**				
MACE, *n* (%)	176 (39.8)	86 (32.1)	90 (51.7)	<0.001
Mortality, *n* (%)	72 (16.5)	29 (10.9)	43 (25.1)	<0.001
Dialysis, *n* (%)	17 (4.0)	5 (1.9)	12 (7.5)	0.004
Re-hospitalization, *n* (%)	97 (23.7)	55 (21.2)	42 (28.2)	0.108
TVR, *n* (%)	7 (1.7)	2 (0.8)	5 (3.3)	0.054
Stroke, *n* (%)	7 (1.7)	1 (0.4)	6 (3.9)	0.007

Abbreviations: CI-AKI, contrast-induced acute kidney injury; WHF, worsening heart failure; MACE, major adverse clinical events; AMI, acute myocardial infarction; TVR, target vessel revascularization.

The patients in the higher HV/W group showed higher rates of mortality and major adverse clinical events (MACE) than the lower HV/W group (2-year mortality: 24.6% vs 9.1%, *P* < 0.001; 3-year mortality: 25.1% vs 10.9%, *P* < 0.001; 2-year MACE: 46.0% vs 26.5%, *P* < 0.001; 3-year MACE: 51.7% vs 32.1%, *P* < 0.001). However, re-hospitalization rates were similar between the HV/W > 15 mL/kg and HV/W ≤ 15 mL/kg groups (2-year re-hospitalization: 21.5% vs 17.3%, *P* = 0.299; 3-year re-hospitalization: 28.2% vs 21.2%, *P* = 0.108) (Table 2).

### Logistic regression analysis for association between HV/W ( > 15 mL/kg) and CI-AKI and WHF

Multivariate logistic regression analysis revealed that compared with HV/W ≤ 15 mL/kg, HV/W > 15 mL/kg was an independent predictor of CI-AKI (odds ratio [OR], 2.33; 95% confidence interval [CI], 1.26–4.31; *P* = 0.016; Table 3) and WHF (OR, 2.13; 95% CI, 1.14–3.99; *P* = 0.018; Table 4).

**Table 3:** Univariate analyses and multivariate associations between contrast-induced acute kidney injury and a hydration volume-to-body weight ratio (HV/W > 15 vs. HV/W ≤ 15 mL/kg)

Risk factors	Univariate logistic regression			Multivariate logistic regression		
	OR	95% CI	*P*-value	OR	95% CI	*P*-value
HV/W >15 mL/kg	2.88	1.82–4.57	<0.001	2.33	1.26–4.31	0.007
Age	1.05	1.03–1.08	<0.001	1.04	1.01–1.08	0.023
CrCl	0.98	0.97–0.99	<0.001	1.01	0.99–1.02	0.227
Diabetes mellitus	1.15	0.71–1.85	0.578	1.18	0.63–2.21	0.602
Anemia	1.53	0.97–2.40	0.067	1.49	0.78–2.82	0.225
Use of IABP	6.71	3.90–11.55	<0.001	4.83	2.31–10.12	<0.001
Use of diuretic	2.04	1.28–3.24	0.003	1.48	0.80–2.74	0.210
Coronary lesion	1.40	1.09–1.80	0.009	1.31	0.97–1.76	0.075
Emergency PCI	2.92	1.84–4.61	<0.001	3.55	1.80–7.01	<0.001
Hypertension	3.51	1.72–7.16	0.001	0.37	0.08–1.73	0.205

Abbreviations: OR, odds ratio; CI, confidence interval; CrCl, creatinine clearance; IABP, intra-aortic balloon pump; PCI, percutaneous coronary intervention.

**Table 4:** Univariate analyses and multivariate associations between worsening heart failure and hydration volume-to-body weight ratio (HV/W > 15 vs. HV/W ≤ 15 mL/kg)

Risk factors	Univariate logistic regression			Multivariate logistic regression		
	OR	95% CI	*P*-value	OR	95% CI	*P*-value
HV/W >15 mL/kg	3.26	1.82–5.85	<0.001	2.13	1.14–3.99	0.018
Age	1.03	1.01–1.06	0.017	1.02	0.99–1.05	0.265
Anemia	1.38	0.79–2.43	0.261	1.18	0.64–2.20	0.594
Use of IABP	4.14	2.20–7.80	<0.001	2.45	1.24–4.84	0.010
Use of diuretic	3.81	2.00–7.28	<0.001	2.82	1.43–5.57	0.003
Emergency PCI	4.23	2.37–7.55	<0.001	3.38	1.81–6.31	< 0.001

Abbreviations: OR, odds ratio; CI, confidence interval; IABP, intra-aortic balloon pump; PCI, percutaneous coronary intervention.

### Long-term mortality according to CI-AKI and WHF

Long-term mortality was significantly higher in patients with CI-AKI than in patients without CI-AKI (41.4% vs 11.4%, *P* < 0.001). Long-term mortality was also higher in patients with WHF than in patients without WHF (45.8% vs 12.9%, *P* < 0.001); the same trend was noted for MACE (CI-AKI: 61.6% vs 35.1%, *P* < 0.001; WHF: 63.2% vs 37.0%, *P* < 0.001) (Supplementary Figure 2). Kaplan–Meier curve analyses revealed that CI-AKI and WHF were significantly associated with an increased risk of mortality (CI-AKI alone: *P* < 0.001; WHF alone: *P* < 0.001; CI-AKI and WHF: *P* < 0.001, (Supplementary Figure 3).

### Cox regression analysis of the association between CI-AKI and WHF and long-term mortality

**Figure 3:**
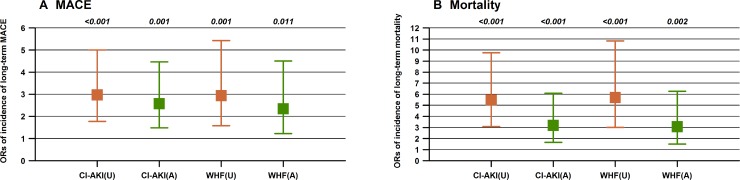
Unadjusted (U) and adjusted (A) odds ratios for long-term mortality (Panel **B**) and MACE (Panel **A**) among patients with CI-AKI or WHF.

**Figure 4:**
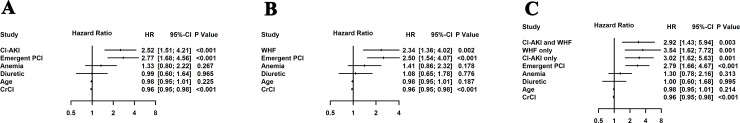
Adjusted hazard ratios of Cox analysis for mortality of CI-AKI vs. No CI-AKI (**A**), WHF vs. No WHF (**B**), and CI-AKI and WHF combined groups (**C**). CI-AKI, contrast-induced acute kidney injury; WHF, worsening heart failure.

Multivariate Cox regression analysis revealed that CI-AKI (hazard ratio [HR] 2.52; 95% CI 1.51–4.21; *P* < 0.001) and WHF (HR 2.34; 95% CI 1.36–4.02; *P* = 0.002;) were significantly associated with mortality, after adjusting for confounding clinical factors including age, anemia, creatinine clearance, diuretics, and emergency PCI; the same trend was noted for MACE (Figure 3). In addition, the combination of CI-AKI with WHF was also found to increase the risk of mortality (HR 2.92; 95% CI 1.43–5.94; *P* = 0.003), after adjusting for confounding clinical factors (Figure 4).

## DISCUSSION

To our knowledge, this is the first study to explore the optimal hydration volume for the prevention of CI-AKI after CAG or PCI among patients with advanced CHF. Our data showed that high hydration volumes (HV/W > 15 mL/kg) were significantly associated with an increased risk of CI-AKI and WHF. Moreover, CI-AKI and WHF were significantly associated with an increased risk of long-term mortality after CAG.

Current studies have shown a lack of consistency regarding the optimal hydration volume and duration for preventing CI-AKI, especially in patients with CHF patients [13]. The 2010 European Guidelines recommend hydration at a rate of 0.5 mL/kg/h for 12 hours before and 24 hours afterward for patients with CHF and chronic kidney disease; however, this recommendation was not supported with clinical evidence [5]. The 2014 ESC guidelines recommend a reduction of the hydration volume from the initial dose of 250 mL, generally recommended for patients, to 150 mL (over 30 minutes) in patients with kidney disease and left ventricular dysfunction (Class IIb) [4]. Marenzi *et al*. recommended hydration with normal saline (0.9%) at a rate of 0.5 ml/kg/h for 12 hours among patients with overt heart failure or left ventricular dysfunction (ejection fraction < 40%) undergoing primary PCI [7]. Our data in the present study showed that a higher hydration volume increased the risk of CI-AKI and WHF. A hydration volume-to-weight ratio less than 15 mL/kg, equal to hydration for 30 hours at 0.5 mL/kg/h, seemed associated with a reduced risk of CI-AKI or WHF following CAG or PCI among patients with advanced CHF.

Iatrogenic hyper-hydration may lead to fluid overload, which results in renal tissue edema, impaired oxygen and metabolite diffusion, distorted renal tissue architecture, and obstruction of capillary blood flow and lymphatic drainage; contributing to the progression of CI-AKI [8, 14]. Fluid overload may also worsen intra-abdominal hypertension, particularly in critically ill trauma or burn patients, leading to further reductions in renal blood flow, venous outflow, renal perfusion pressure, and urine output. These outcomes are, in turn, strongly associated with the development of CI-AKI [15].

Multiple data consistently demonstrate that fluid overload plays a central role in the pathogenesis of WHF and patient hospitalization. Fluid overload and increased ventricular volumes, therefore, foster left ventricular remodeling and mitral regurgitation both directly, through myocardial stretch and indirectly, through the activation of the renin-angiotensin-aldosterone, adrenergic, and cytokine systems [16, 17]. Fluid overload and increased intraventricular pressure also cause coronary hypoperfusion and subendocardial ischemia, which may further impair cardiac function [18]. A recent study showed that central venous pressure (CVP)-guided hydration can safely and effectively reduce the risk of CI-AKI in patients with chronic kidney disease and CHF, without increasing the incidence of acute heart failure [19]. However, CVP measurement is invasive and its use as a routine procedure in clinical practice is infeasible.

In the present study, we found that CI-AKI was significantly associated with long-term mortality, which is in general agreement with the results of our previous study [20, 21]. A meta-analysis by James *et al*. investigated the association between CI-AKI after CAG and adverse clinical outcomes [22]. Of the 34 included studies examining mortality, 33 reported significantly increased mortality rates in patients who developed CI-AKI after CAG. The pooled adjusted risk ratio for mortality and cardiovascular events was 2.39 and 1.98, respectively, after adjustment for confounders in 23 studies.

WHF was also significantly associated with long-term mortality in the present study. Similarly, a previous study found that patients who experienced WHF had markedly worse clinical outcomes at 30 and 180 days [3]. By 180 days, 41.5% of patients with WHF had died compared with 11.3% of patients without WHF. In another study assessing patients who were admitted to a hospital for acute heart failure, WHF occurred in 27% patients and was associated with prolonged hospitalization and higher readmission and death rates [23]. However, few studies have focused on WHF and prognosis in patients with advanced CHF undergoing CAG. Our study provides strong evidence over a long follow-up period (2.48 years) that in patients with advanced CHF undergoing CAG, WHF also significantly increases the risk of long-term MACE.

Recently, the AMACING trial investigated the effect of hydration on the risk of contrast-induced nephropathy (CIN) and cost among patients receiving contrast exposure, and showed that no-hydration was non-inferior and cost-saving for preventing contrast-induced nephropathy compared with intravenous hydration recommended by current clinical practice guidelines [4, 8, 24, 25]. Although no data of baseline heart function (history of heart failure, left ventricular ejection fraction, heart function classification, or other biomarkers) were available in the AMACING study, the guidelines hydration protocol increased the risk of post-hydration symptomatic heart failure (4% vs 0%, suggesting the importance of safe hydration for preventing CI-AKI, especially among patients with pre-existing advanced heart failure.

### Study limitations

The present study has several limitations. First, our study was a single-center prospective observational study with a limited sample size. The results from this hypothesis-generating study need further assessment in a randomized controlled trial. Second, variation in the measurement times may have led to the missing of post-procedural peak levels of creatinine. Thirdly, there may have been some bias in the final hydration volume, as cardiologists may administer higher hydration volumes for patients with more baseline risk factors for CI-AKI and WHF. This, in turn, may have confounded the results regarding the effect of hydration volume on the risk of CI-AKI and WHF, despite the adjustment for confounders. Fourthly, although we previously set up the same hydration speed for those patients, physicians or nurses involved in the management of some patients may have adjusted the final hydration speed, which was also an unadjusted-for confounder. These possible variations of the hydration protocol owing to the treating physician and nurses may have significantly affected the study results. Finally, data was lacking regarding oral water intake and we were unable to investigate whether patients with low hydration volumes might have drunk more water (higher oral hydration volume) and had reached the same or similar hydration volumes as those treated with higher intravenous hydration volumes.

## METHODS

### Subjects

In this prospective observational study, we enrolled patients who were candidates for CAG or PCI between January 2010 and October 2012, according to the institutional protocol. As per our sub-study in a previous publication [12], we included patients aged ≥18 years who had advanced CHF (NYHA class > 2) or a history of pulmonary edema [2, 26]. As per the updated European Society of Urogenital Radiology Contrast Media Safety Committee guidelines [27], the exclusion criteria included pregnancy, lactation, intravascular administration of contrast medium within 7 days before or 3 days after the procedure), non-use of low-osmolarity contrast agents, cardiovascular surgery or endovascular repair, end-stage renal disease or renal replacement, missing pre- or postoperative creatinine values, malignancy, non-use of isotonic saline for hydration, and patients without advanced CHF (Supplementary Figure 1).

Follow-up events were carefully monitored and recorded by trained nurses through office visits and telephone interviews at 1, 6, 12, 24, 36, and 48 months after CAG. The mean follow-up time was 2.62 ± 0.9 years (median, 2.48; interquartile range [IQR], 1.89–3.45 years). The investigation complied with the principles outlined in the Declaration of Helsinki and was approved by the Ethics Committee of the Guangdong General Hospital. All patients gave their written informed consent.

### Coronary angiography

Cardiac catheterization was performed according to standard clinical practice [9]. All patients received non-ionic, low-osmolarity contrast agents. Patients were treated based on recent guidelines [27]. All patients received a continuous intravenous infusion of isotonic saline at a rate of 0.5 mL/kg/h for at least 2 to 12 hours before the procedure and continued to receive it 6 to 24 hours afterward. Serum creatinine concentrations were also measured in accordance with our clinical protocol [9] in all patients on admission to the hospital and on day 1, 2, and 3 after the procedure. Creatinine clearance was calculated using the Cockcroft–Gault formula [28], and hydration volume/weight (HV/W, mL/kg) ratios were calculated.

### Endpoints and definitions

The primary endpoint was the first occurrence of CI-AKI and WHF, and the secondary endpoint was the first occurrence of any MACE during follow-up. CI-AKI was defined as an increase in serum creatinine of ≥0.3 mg/dL or ≥50% from baseline within 48 hours of contrast exposure [29]. WHF was defined as the presence of at least one sign, symptom, or radiologic indication of new, persistent, or worsening acute heart failure requiring the addition of new intravenous therapy (inotrope or vasodilator) or mechanical support during a patient's index hospitalization targeted specifically at heart failure symptoms [3]. MACE was defined as mortality, re-non-fatal acute myocardial infarction, target vessel revascularization, CI-AKI requiring renal replacement therapy, stroke, and re-hospitalization after the index hospitalization.

### Statistical analysis

For continuous variables, two independent sample *t*-tests were performed for normally distributed data (presented as mean ± standard deviation), and the Wilcoxon rank-sum test was used for non-normal distributions (presented as median and IQR). Pearson's chi-square test or Fisher's exact test were used as appropriate for categorical data, which are expressed as percentages. The Cochran-Armitage test was performed to investigate the association between HV/W and the incidence of CI-AKI and WHF. ROC curves were used to identify the optimal sensitivity for the observed range of HV/W for CI-AKI.

After balancing overfitting and the good control of unbalanced factors, we used factors with *P*-values < 0.05 at baseline analysis along with clinically important factors for the multivariate logistic analysis to ascertain the ability of HV/W (> 15 mL/kg vs. ≤ 15 mL/kg) to predict CI-AKI and WHF. Univariate analyses of mortality were performed using the log-rank test, and multivariate Cox regression analyses adjusting for the use of an intra-aortic balloon pump, anemia, diabetes mellitus, emergency PCI, age (> 75 years), and for other factors were also performed.

The data were analyzed on an available case basis, and missing data were not included. All data analyses were performed using SAS version 9.4 (SAS Institute, Cary, NC, USA) and R software (version 3.1.2, R Core Team, Vienna, Austria) [30]. Two-sided *P*-values < 0.05 were considered statistically significant.

## CONCLUSIONS

Our data showed that higher hydration volumes were associated with a significantly increased risk of CI-AKI and WHF after CAG in high-risk patients with advanced CHF. The relatively safe hydration volume for patients with advanced CHF might be as low as 15 mL/kg. Moreover, CI-AKI and WHF were associated with a significantly increased risk of long-term mortality. The potential renal and heart safety benefits of a personalized control of hydration volume in patients with advanced CHF need to be investigated in further large-scale multicenter randomized controlled trials.

## SUPPLEMENTARY MATERIALS FIGURES



## Abbreviations

CAG: coronary angiography
CHF: congestive heart failure
CI: confidence interval
CI-AKI: contrast-induced acute kidney injury
CVP: central venous pressure
HR: hazard ratio
HV/W: hydration volume-to-weight ratio
IQR: interquartile range
MACE: major adverse clinical events
NYHA: New York Heart Association
OR: odds ratio
PCI: percutaneous coronary intervention
ROC: receiver operating characteristic
WHF: worsening heart failure
