# Supplementary material for: Optimal hydration volume among high-risk patients with advanced congestive heart failure undergoing coronary angiography
Source: Oncotarget. 2018 May 4;9(34):23738–48. doi: 10.18632/oncotarget.25315 (PMC5955121; doi:10.18632/oncotarget.25315)
Supplement: Supplementary file 1 [file oncotarget-09-23738-s001.pdf]

Optimal hydration volume among high-risk patients with advanced congestive heart failure undergoing coronary angiography

SUPPLEMENTARY MATERIALS

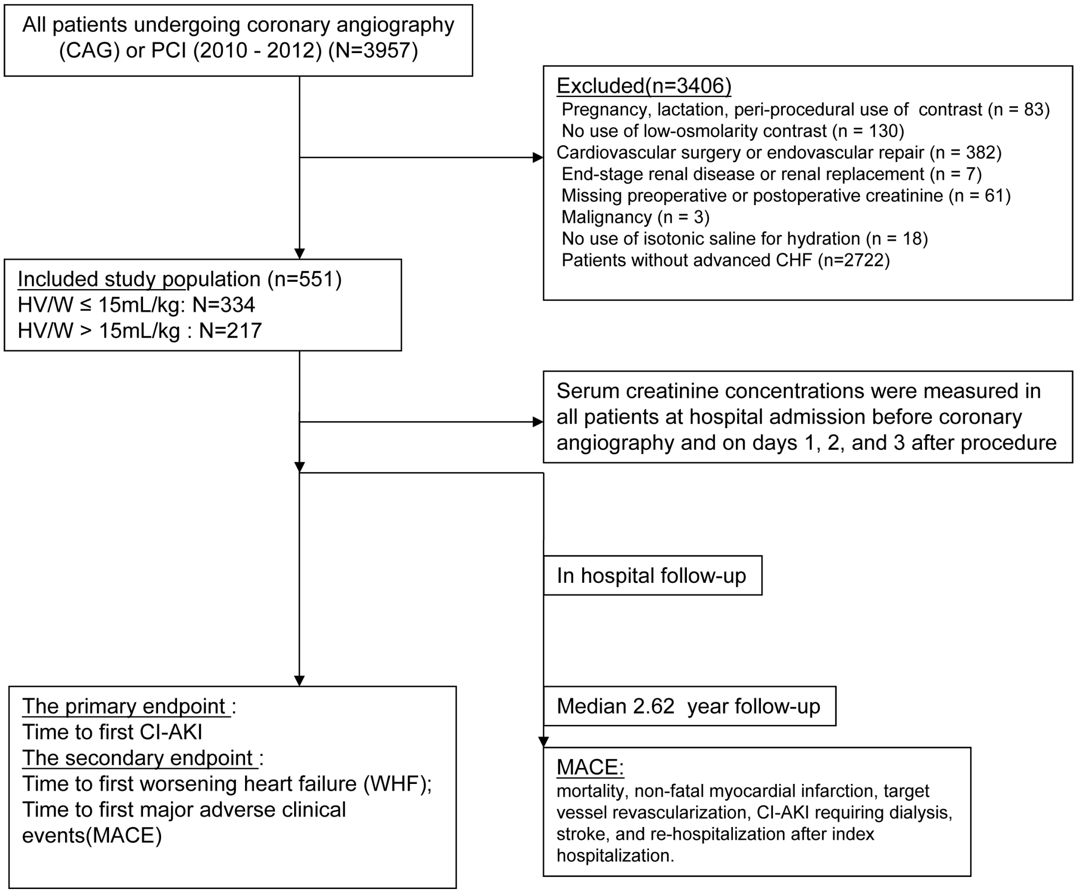

Supplementary Figure 1: Study flow.

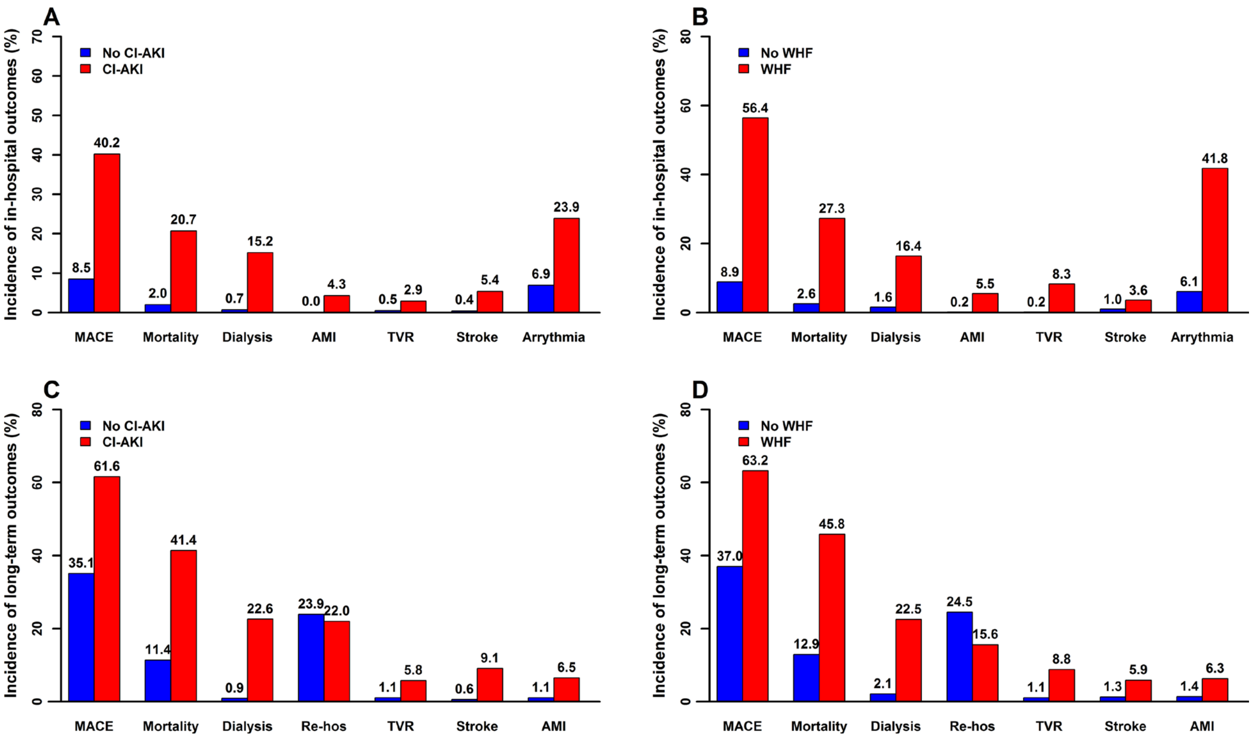

Supplementary Figure 2: Association of CI-AKI and WHF with mortality and MACE.

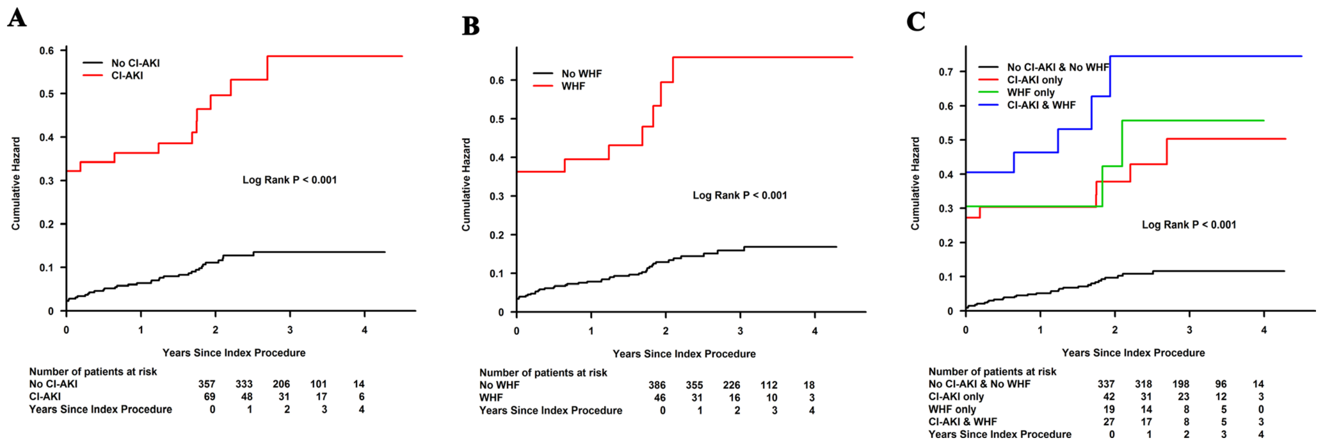

Supplementary Figure 3: Kaplan–Meier curve analyses according to CI-AKI and WHF.
